# Cardiac Biomarkers in the Emergency Department: The Role of Soluble ST2 (sST2) in Acute Heart Failure and Acute Coronary Syndrome—There is Meat on the Bone

**DOI:** 10.3390/jcm8020270

**Published:** 2019-02-22

**Authors:** Aneta Aleksova, Alessia Paldino, Antonio Paolo Beltrami, Laura Padoan, Massimo Iacoviello, Gianfranco Sinagra, Michele Emdin, Alan S. Maisel

**Affiliations:** 1 Cardiovascular Department, Azienda Sanitaria Universitaria di Trieste and Department of Medical Surgical and Health Sciences, University of Trieste, 34100 Trieste, Italy; alessiapaldino@gmail.com (A.P.); gianfranco.sinagra@asuits.sanita.fvg.it (G.S.); 2 Department of Medicine, University of Udine, 33100 Udine, Italy; antonio.beltrami@uniud.it; 3 Sport and Exercise Medicine Division, Department of Medicine, University of Padova, 35122 Padova, Italy; argonauta92@hotmail.it; 4 University Cardiology Unit, Cardiothoracic Department, University Policlinic Hospital of Bari, 70124 Bari, Italy; massimo.iacoviello@gmail.com; 5 Department of Life science, Scuola Superiore Sant’Anna, 56127 Pisa, Italy; emdin@ftgm.it; 6 Fondazione Toscana Gabriele Monasterio, National Research Council, 56124 Pisa, Italy; 7 Division of Cardiology, University of California San Diego, San Diego, CA 92093, USA; asmaisel@gmail.com

**Keywords:** sST2, biomarkers, stratification, myocardial infarction, acute heart failure, emergency department

## Abstract

Soluble ST2 (sST2) has recently emerged as a promising biomarker in the field of acute cardiovascular diseases. Several clinical studies have demonstrated a significant link between sST2 values and patients’ outcome. Further, it has been found that higher levels of sST2 are associated with an increased risk of adverse left ventricular remodeling. Therefore, sST2 could represent a useful tool that could help the risk stratification and diagnostic and therapeutic work-up of patients admitted to an emergency department. With this review, based on recent literature, we have built sST2-assisted flowcharts applicable to three very common clinical scenarios of the emergency department: Acute heart failure, type 1, and type 2 acute myocardial infarction. In particular, we combined sST2 levels together with clinical and instrumental evaluation in order to offer a practical tool for emergency medicine physicians.

## 1. Introduction

ST2 is a member of the superfamily of interleukin (IL)-1 receptors that exists in two forms: A transmembrane receptor (ST2L) and a soluble one (sST2, denoted as ST2), expressed through an alternative splicing [1]. The natural ligand of ST2 is IL-33, a member of the IL-1 family, which can act both as a traditional cytokine and as a transcription factor [2]. IL-33 is produced by distinct cell types that act as a barrier (e.g., quiescent endothelial cells, lung and gut epithelial cells, keratinocytes, fibroblasts, and smooth muscle cells) and is downregulated under inflammatory conditions [2]. In intact cells, IL-33 shows a nuclear localization and acts as a transcriptional regulator that regulates, among others, nuclear factor NFκB transcriptional activity [2]. However, following cell injury or necrotic cell death, IL-33 is released from the cell and functions as an alarmin [2]. Once released, IL-33 orchestrates the immune response, especially natural immunity [2]. Because of the complex actions that IL-33 plays in tissue injury and inflammation, it has been involved in the pathogenesis of several diseases (e.g., allergy, autoimmune diseases, cancer, atherosclerosis, and diabetes). Most importantly, IL-33 plays a cardioprotective role, preventing cardiac fibrosis and hypertrophy in response to mechanical load via ST2L [3,4]. Indeed, IL-33 may exert protective effects against atherosclerosis in ApoE^-/-^ mice and prevents adipose tissue inflammation in obese mice [5,6]. Furthermore, the genetic ablation of IL-33 exacerbates cardiac remodeling and impairs cardiac function in mice with heart failure secondary to transverse aortic constriction [7]. Concerning ST2L, it has been shown that its genetic deletion delays wound healing, impairing angiogenesis and promoting macrophage polarization towards a proinflammatory phenotype [8]. sST2, instead, is a decoy receptor, which reduces the cardioprotective effect of IL-33/ST2L pathway by binding free IL-33 [4].

The soluble variant of ST2 results in being overexpressed in specific pathologic conditions of myocardial stress or injury and is associated with inflammation and immune response [9].

Recent observations suggest an important prognostic value of sST2, either in chronic heart failure, where it predicts patients outcome beyond N-terminal pro-B-type natriuretic peptide (NT-proBNP) and high-sensitivity troponin T [10], or in acute heart failure [11], where it is useful for monitoring and driving therapeutic decisions in patients with acutely decompensated heart failure (ADHF) and acute myocardial infarction (AMI) [12].

This review offers some possible therapeutic decision flowcharts for acute cardiovascular diseases, based on the evaluation of sST2 levels in the emergency department.

## 2. sST2 in Acute Decompensated Heart Failure (ADHF)

Acute dyspnea is a common complaint in the Emergency Department (ED) that is complicated by its wide spectrum of possible differential diagnoses. Indeed, the emergency clinician frequently has to distinguish ADHF from the other causes of dyspnea.

Heart failure (HF) is currently one of the main public health problems, affecting at least 26 million people worldwide [13]. The constant growth of HF prevalence, due to the aging of the population, together with the recurrence of acute exacerbations result in an increase in the hospitalization rates and are the main reasons for the high cost of this disease for the healthcare system [14]. Almost 1–2% of the healthcare budget every year is spent by European Countries and the USA on HF [15]. In this context, new strategies of both cost-effectiveness and preventive measures for cardiovascular disease are needed.

sST2, reflecting pathophysiological processes that link the inflammatory and neurohormonal systems, is a promising biomarker in the field of HF that could guide clinicians in the identification of patients at a high risk for ADHF necessitating hospitalization. Further, sST2 could be used as a guide for therapeutic strategies [16]. In addition to natriuretic peptides (NPs), and even independently from other biomarkers, several studies have shown the prognostic utility of sST2 for ADHF [17,18,19]. Indeed, the American Heart Association/American College of Cardiology (AHA/ACC) Guidelines for the Management of HF recommend (class IIb, Level of Evidence B) the measurement of sST2 in patients with ADHF, for a more appropriate risk stratification [20]. Furthermore, in contrast to NPs, sST2 is not influenced by either age, body mass index, renal function, or etiology of HF [21,22] and, compared to the other biomarkers, it has the lowest intra-individual variation and the smallest relative change value [23,24]. For these properties, it has also been tested as a potential biomarker for the differential diagnosis of ADHF [17,25]. The PRIDE (Pro-Brain Natriuretic Peptide Investigation of Dyspnea in the Emergency Department) was the first study that measured sST2 in patients with acute dyspnea [17]. In a population of 593 patients presenting to the ED with an acute onset of dyspnea, the group with ADHF had a concentration of sST2 significantly higher than those without (*p* < 0.001) [17]. Thereafter, the role of sST2 in ruling out ADHF in ED has also been confirmed by Henry-Okafor and colleagues [26]. They reported an area under the curve (AUC) for sST2 that was similar to the PRIDE AUC: 0.62 (95% CI 0.56–0.69) vs. 0.74 (95% CI 0.70–0.78), respectively, but both were inferior to those reported for NPs, in the respective cohorts [17,26,27]. Therefore, whilst sST2 cannot replace NPs for the ADHF diagnosis, it has been demonstrated that sST2 at admission is superior over NT-proBNP in predicting one-year mortality in ADHF patients, especially of those patients with elevated NPs [28]. Januzzi et al. proposed a cutoff value of sST2 ≥ 35 ng/mL as a predictor for worse prognosis in patients with acute HF [29].

**Figure 1:**
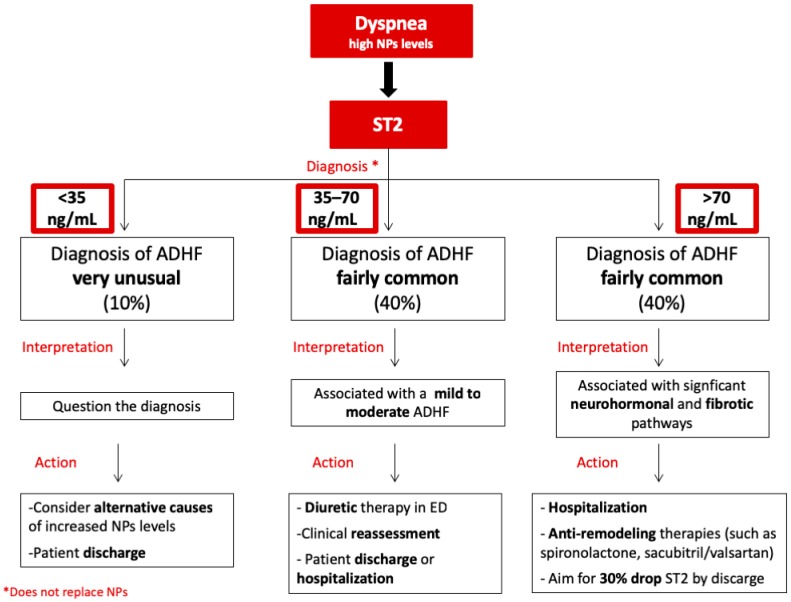
Flowchart showing the proposed soluble ST2 (sST2) aided management of patients with dyspnea and elevated natriuretic peptides (NPs). In patients with dyspnea and elevated NPs, sST2 levels can help to identify 3 classes of patients. If sST2 < 35 ng/mL, the diagnosis of acute decompensated heart failure (ADHF) is unusual. In patients with 35 ≤ sST2 ≤ 70 ng/mL, ADHF is more common but mild to moderate. If sST2 > 70 ng/mL, ADHF is fairly common, requiring hospitalization and anti-remodeling therapies. Suggested actions for each class of patients are shown in the panels below.

In patients with dyspnea and elevated NPs, a sST2 flowchart (Figure 1) could be useful for a more accurate diagnosis, risk stratification, and appropriate treatment of ADHF in the ED, as it has been proposed for chronic heart failure [10]. Literature data showed that dyspneic patients judged as having ADHF had a higher median sST2 value (approximately 70 ng/mL) than those without ADHF [19,30,31,32]. A level of 70 ng/mL would, therefore, constitute a potential cut-off value to distinguish ED patients with a very high risk of ADHF [20]. Indeed, sST2 values above approximately 70 ng/mL have been associated with higher risk of death on both a short- (30 days) and long-term (one year) follow-up [19,30,31,32]. A significant activation of the neurohormonal and fibrotic pathways, which induce an adverse myocardial remodeling after an acute event, could be the reason for these findings [25]. For the high risk of mortality and negative outcomes, hospital admission should be mandatory in this category of patients. Moreover, in high-risk patients, particularly if reduced ejection fraction (EF) is present, anti-remodeling therapies, such as spironolactone and eplerenone, should be administered [12]. In this context, sacubitril/valsartan indication should also be taken into account in the next future [33]. Indeed, in the setting of acute HF, it has been shown that the serial measurement of sST2 levels and the assessment of the dynamic variation of this biomarker during hospitalization are endowed with prognostic implications [24,25]. Van Vark et al. demonstrated that the slope of sST2 level trajectories may also be an independent predictor of their primary endpoint, a composite of all-cause mortality and readmission for HF [34]. The U-shape sST2 pattern (a secondary increase of sST2 level after an initial decrease) seemed to be a predictor of endpoint events, whereas patients with a J-shape sST2 pattern (only an initial decrease of sST2 level) never remained event-free [34]. Consistently, patients with a rapid decrease in sST2 concentrations after hospital admission, in particular with a >30% decrease in sST2 levels from baseline to 48–72 h [35], show an uncomplicated short-term follow-up, as opposed to that of patients with persistently high levels of sST2 [35,36,37]. In this context, the drop of almost 30% in sST2 levels during hospitalization, accompanied by clinical and hemodynamics data, can support the clinician’s decision for discharge. Conversely, sST2 levels <35 ng/mL (Figure 1) have been found in less than 10% of ADHF in ED [34]. That is why in these patients, the diagnosis of ADHF should be questioned and the clinician should search for alternative causes of NPs. The intermediate zone of dyspneic patients and elevated NPs, with sST2 levels comprising between 35 and 70 ng/mL (Figure 1), could therefore contribute to classify, in the ED, mild-to-moderate ADHF. This last category of patients could benefit from the administration of diuretics directly in the ED. If improvement of dyspnea is observed after diuretic therapy, clinical reassessment and instrumental evaluation in ED should be performed, in order to decide if the patient needs hospitalization or could be managed on an outpatient basis.

## 3. ST2 in Acute Myocardial Infarction

### 3.1. sST2 and Type 1 Myocardial Infarction

Myocardial infarction is a major cause of morbidity and mortality worldwide. Type 1 acute myocardial infarction (type 1 AMI) is related to a coronary plaque rupture, or dissection with intraluminal thrombosis and consequent perfusion imbalance between demand and supply. After an AMI, cardiomyocyte necrosis and myocardial fibrosis induce a geometrical and biomechanical modification of the cardiac structure, leading to the process of cardiac remodeling, which is coupled with functional impairment [38]. The viable myocardium surrounding the necrotic and fibrotic areas appears to be subjected to an increased workload and wall tension [39]. Hypertrophy and dilatation are the main myocardial responses to these pathophysiological changes following AMI [40,41]. In this context, cardiomyocyte necrosis and mechanical stretching cause the release of some biomarkers, respectively, troponin and NPs [42]. Additionally, sST2 was presumed to be secreted by myocardial cells (cardiomyocytes and fibroblasts) in AMI as a consequence of cardiac overload [39,43]. However, most recent data seem to identify in vascular endothelial cells one of the main sources of sST2 [44]. These features have suggested that sST2 may act as biomarker of vascular health [45], providing multiple information on the pathophysiological state of AMI patients that should be taken into account in association with troponin level assessment. Therefore, circulating sST2 levels, already increased one day after AMI [43], have been studied for their prognostic function. The early detection of high-risk AMI patients is intended to reduce worst disease evolution and negative outcomes [46]. Indeed, different, large clinical trials have demonstrated the prognostic significance of sST2 on a short-term (30 days) follow-up of patients with ST elevation myocardial infarction (STEMI). Specifically, Shimpo et al. measured circulating sST2 levels of 810 patients with STEMI that were enrolled in the thrombolysis in myocardial infarction (TIMI) 14 and enoxaparin and TNK-tPA with or without GPIIb/IIIa inhibitor as reperfusion strategy in STEMI (ENTIRE)-TIMI 23 clinical trials [47]. Higher early levels of sST2 have been correlated with mortality and with the development of new or worsening of congestive HF by 30 days after STEMI. In most of these patients, the sST2 peak values occurred at 12 h. In contrast to NT-proBNP that increases exponentially in four days after STEMI, the sST2 12 hour peak is followed by a significant reduction [47]. Furthermore, early sST2 levels were not associated with age, hypertension or previous MI, which increase left ventricular (LV) wall stress, compared to NT-proBNP [48]. These are the reasons why early sST2 levels are more predictive of cardiovascular outcomes than NPs. However, in a subgroup of the clopidogrel as adjunctive reperfusion therapy-thrombolysis in myocardial infarction 28 (CLARITY-TIMI 28) trial, involving STEMI patients, Sabatine et al. created a multimarker model with sST2, NT-proBNP, and traditional cardiovascular risk factors. Indeed, they demonstrated that both biomarkers remain independent predictors of cardiovascular death and HF [48]. In addition, the inclusion of both sST2 and NT-proBNP to the TIMI risk score showed an improvement of c statistics from 0.73 (95% CI, 0.68–0.78) to 0.78 (95% CI, 0.74–0.83; *p* = 0.0025), resulting in a more precise risk stratification [48]. sST2 levels in NSTEMI patients parallel the behavior observed in STEMI. Currently, few studies have assessed the clinical implication of increased sST2 levels in this context. Kohli et al. showed that NSTEMI patients enrolled in the metabolic efficiency with ranolazine for less ischemia in non ST-elevation acute coronary syndrome thrombolysis in myocardial infarction 36 (MERLIN-TIMI 36) trial presented an acute elevation of sST2 [49]. At 30 days after AMI, early sST2 elevation >35 ng/mL was associated with a higher risk for cardiovascular death and HF. These results were confirmed at one year. An integration of different biomarkers (i.e., sST2, troponin, NPs) with clinical characteristics seems to improve risk stratification, such as for STEMI patients [49].

**Figure 2:**
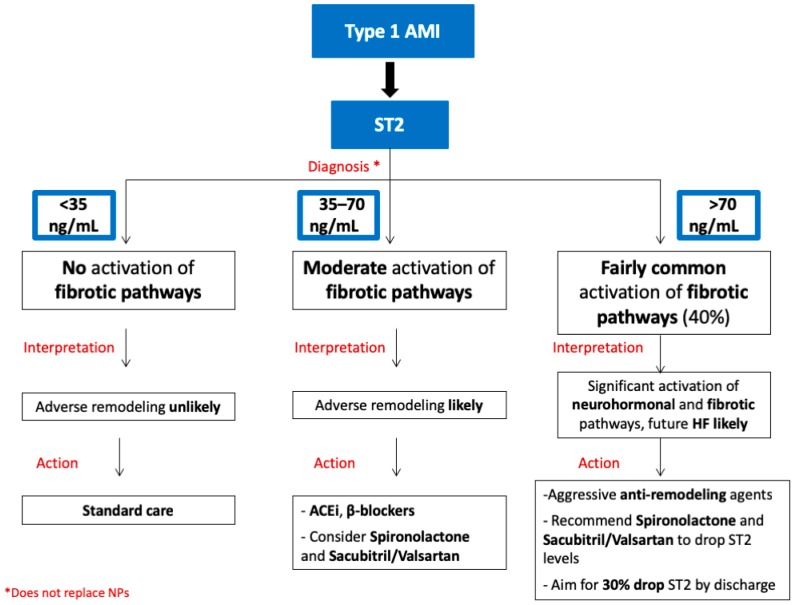
Flowchart summarizing soluble ST2 (sST2) aided therapeutic decision-making for patients with type 1 myocardial infarction. In patients with type 1 acute myocardial infarction (AMI), sST2 levels can help to identify 3 classes of patients. If sST2 <35 ng/mL, adverse remodeling is unlikely. In patients with 35 ≤ sST2 ≤ 70 ng/mL, adverse remodeling is more likely, and patients could benefit from antifibrotic therapies. If sST2 >70 ng/mL, adverse remodeling is fairly common, requiring aggressive anti-remodeling therapies. Suggested actions for each class of patients are shown in the panels below.

Therefore, even if sST2 does not add anything to the initial diagnosis of AMI, its prognostic role has become remarkable. Actually, early sST2 levels in AMI seem to reflect the extent of myocardial necrosis, given its negative correlation with LV ejection fraction at 1 day after AMI [43]. Furthermore, the inverse correlation between baseline sST2 and LV ejection fraction was also confirmed 24 weeks after AMI [50]. From all these literature data, sST2 could be recognized as a marker of early and late post-infarct remodeling. Therefore, consistently with what we discussed in the AHF setting, the measurement of sST2 in patients with a diagnosis of AMI could lead to specific therapeutic decisions, based on the assessment of sST2 levels in the ED (Figure 2).

The research group of Jenkis et al. were able to divide a cohort of 1401 patients with incident MI into 3 cardiovascular risk classes on the basis of early sST2 values [51]. Importantly, the 2nd (37 < sST2 ≤ 72.3 ng/mL) and 3rd (sST2 > 72.3 ng/mL) tertiles were associated with a higher risk of mortality during the first 30 days and the first 5 years of follow-up, independently from other clinical prognostic indicators. Furthermore, the authors found only a weak association between sST2 values and maximum troponin levels. As for HF, patients with MI and sST2 > 72.3 ng/mL seemed to be more subject to a significant activation of neurohormonal and profibrotic pathways that induce a significant increased risk of adverse myocardial remodeling and HF. In this case, standard care for AMI should be associated with aggressive anti-remodeling agents, such as spironolactone, if the patient’s potassium levels and renal function allow this approach [50], and, possibly, sacubitril/valsartan. The discharge decision could be guided by the evaluation of sST2 levels during hospitalization, until they decline by at least 25% from admission [12]. While for patients with 0 < sST2 ≤ 35 ng/mL, AMI standard care should be enough, different considerations should be done for the second class of risk. Actually, AMI patients with 35 < sST2 ≤ 70 ng/mL might also have an adverse myocardial remodeling more frequently than those with sST2 ≤ 35 ng/mL (Figure 2). If for them, pharmacological therapy with ACE-inhibitors and beta blockers is necessary, spironolactone and sacubitril/valsartan could be considered as well [12].

### 3.2. sST2 and Type 2 Myocardial Infarction

Among patients presenting to an ED with elevated cardiac troponin and symptoms indicative of acute coronary syndrome, the prevalence of type 2 myocardial infarction (type 2 AMI) has a wide variability, ranging from 1.6–71% [52]. Type 2 AMI more frequently occurs among older patients with numerous comorbid conditions [52,53] and is associated with a poor long-term outcome [54]. In type 2 AMI, a comorbid condition (e.g., sustained tachyarrhythmia, atrial fibrillation (AF), severe hypertension, respiratory failure, severe anemia or shock) is the major contributor of a significant imbalance between myocardial oxygen supply and/or demand with consequent cardiac troponin elevation [55]. More recently, highly sensitive cardiac troponins are causing a “plague of troponinitis” [56]. Therefore, stratification of patients with AMI on the basis of troponin elevation is becoming a complex task for the cardiology community.

In all type 2 AMI conditions, an accurate clinical examination, together with an echocardiographic evaluation, is, generally, a useful diagnostic strategy, crucial for therapeutic decision-making. However, a decisional flow chart for risk stratification and management of type 2 AMI patients in ED could take advantage of sST2 measurement. sST2 is involved in pathophysiology of cardiac fibrosis and inflammation and is a powerful predictor of adverse outcomes [48]. This concept is true in type 2 AMI too, where its levels seem to be associated with future myocardial remodeling and worse outcome [57]. It is well-known that fibrotic and remodeling processes of the atria promote the maintenance and occurrence of AF [58]. In one recent study [57], sST2 levels correlated with left atrial diameter and were more elevated in patients with a more persistent form of AF. Further, sST2 levels in patients with AF are an independent predictor of subsequent HF [57]. Nortamo et al. [59], including patients from the Ambulatory blood pressure Registry TEleMonitoring (ARTEMIS) of hypertension and cardiovascular rISk-ARTEMIS study, found that elevated levels of sST2 (≥24.85 ng/mL) were associated with the risk of occurrence of new-onset AF during long-term follow-up, even after adjustments for the relevant clinical risk factors. Furthermore, recently, it was demonstrated that the cut-off value of sST2 30.6 ng/mL could predict AF recurrence after cryoballoon ablation [60]. Further, sST2 levels have been associated with blood pressure, the use of antihypertensive treatment as well as diabetes mellitus [61], conditions frequently observed in type 2 AMI patients.

**Figure 3:**
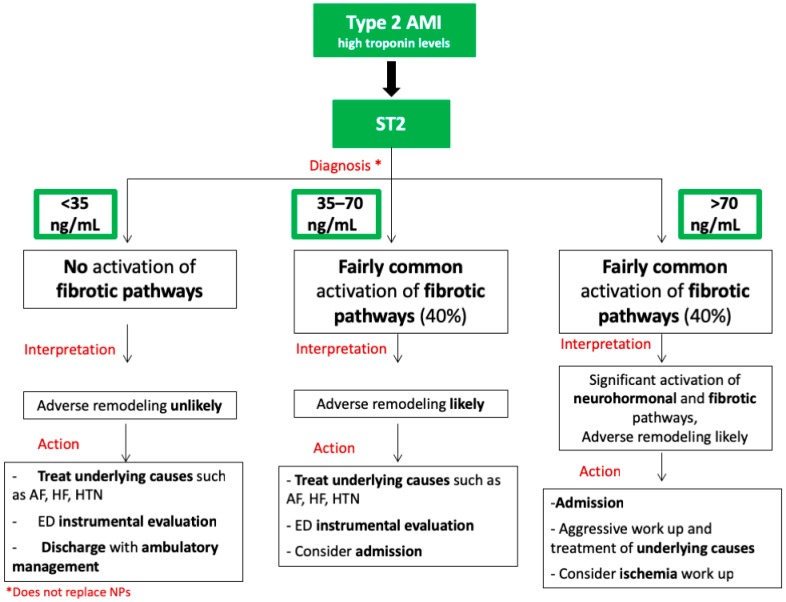
Flowchart summarizing sST2 aided therapeutic decision-making for patients with type 2 myocardial infarction. In patients with type 2 acute myocardial infarction (AMI) and elevated troponin levels, sST2 levels can help to identify 3 classes of patients. If sST2 < 35 ng/mL, adverse remodeling is unlikely. In patients with 35 ≤ sST2 ≤ 70 ng/mL, adverse remodeling is more likely, and patients could benefit from antifibrotic therapies. If sST2 > 70 ng/mL, a significant activation of neurohormonal and fibrotic pathways is likely, requiring aggressive anti-remodeling therapies. Suggested actions for each class of patients are shown in the lower panels.

We propose a flow chart based on sST2 levels for the management of patients with type 2 AMI (Figure 3). It should be taken with caution, since this setting necessitates further investigation, given that only few studies have explored the usefulness of sST2 measurement in this subset of patients. In summary, in all patients with type 2 AMI, acute management should focus on treating the underlying causes in ED (Figure 3). Since an adverse LV remodeling is unlike when sST2 is <35 ng/mL [9], discharge of the patient after acute treatment with further ambulatory management should be considered. Treatment of the underlying cause of troponin elevation in ED and then hospital admission may be necessary in patients with sST2 levels >35 ng/mL. In case of important elevation of sST2 > 70 ng/mL, reflecting the activation of the neurohormonal, inflammatory, and fibrotic pathways [25], an even more aggressive diagnostic and therapeutic work-up should be considered. However, ischemia work-up should be reserved only for cases with high suspicion of the coexistence of a coronary atherosclerotic burden.

## 4. Conclusions

In conclusion, sST2, represents, especially when combined with NPs, troponins, and clinical variables, a promising tool that could improve the risk stratification and diagnostic–therapeutic work-up of patients admitted to the ED. Therefore, in this review, we are suggesting a sST2-assisted patient stratification approach in three clinical scenarios (i.e., ADHF, T1MI, and T2MI), whose clinical usefulness should be validated in larger prospective clinical studies. However, we are confident in our conclusion that sST2 plasma levels above 70 ng/mL seem to be associated with a significant activation of both neurohormonal and fibrotic pathways and can help to identify those patients that are affected by ADHF, type 1, and type 2 AMI that are at increased risk of adverse LV remodeling. Serial assessment of sST2 should also be considered for a better management of patients and more appropriate therapeutic decisions, since clinical data suggest that the kinetics of sST2 plasma levels can discriminate patients with different outcomes.
